# Architecture of *Saccharomyces cerevisiae* SAGA complex

**DOI:** 10.1038/s41421-019-0094-x

**Published:** 2019-05-07

**Authors:** Gaochao Liu, Xiangdong Zheng, Haipeng Guan, Yong Cao, Hongyuan Qu, Junqing Kang, Xiangle Ren, Jianlin Lei, Meng-Qiu Dong, Xueming Li, Haitao Li

**Affiliations:** 1 MOE Key Laboratory of Protein Sciences, Beijing Advanced Innovation Center for Structural Biology, Tsinghua-Peking Joint Center for Life Sciences, School of Life Sciences and School of Medicine, Tsinghua University, Beijing, 100084 China; 2 National Institute of Biological Sciences, Beijing, 102206 China

**Keywords:** Cryoelectron microscopy, Histone post-translational modifications

Dear Editor,

Eukaryotic gene transcription by RNA polymerase II (Pol II) is highly regulated and fine-tuned by diverse epigenetic complexes, which often function as co-activators or co-repressors to modulate transcription in concert with sequence-specific transcription factors (TFs). In Saccharomyces cerevisiae, the 1.8-MDa coactivator SAGA (Spt–Ada–Gcn5–acetyltransferase) complex^1^ was reported to be required for genome-wide transcription by RNA Pol II, and proposed to function as a general cofactor^2^. Yeast SAGA is composed of 19 subunits, which are categorized into four modules, namely HAT (Gcn5, Ada2, Ada3, and Sgf29), DUB (Ubp8, Sgf11, Sgf73, and Sus1), TAF (Taf5, Taf6, Taf9, Taf10, and Taf12), and SPT (Tra1, Ada1, Spt3, Spt7, Spt8, and Spt20). Two catalytic subunits, Gcn5 within HAT^1^ and Ubp8 within DUB^3^, permit histone H3 acetylation and ub-H2B deubiquitylation activities of the SAGA complex. Remarkably, acetylation and deubiquitylation carried out by SAGA are crucial for transcriptional initiation and elongation; in yeast, inactivation of SAGA leads to a global decrease of RNA Pol II-mediated transcription^2^.

The largest SAGA component Tra1 (3744 residues) in the SPT module was shown to directly interact with the acidic activation domain of TFs, such as VP16, GCN4, Gal4, and HAP4^4^, which help recruit SAGA to promoter regions of target genes. Tra1 is also a subunit of the yeast NuA4 acetyltransferase complex^5^. Roles of Tra1 in SAGA and NuA4 assembly, as well as TF binding await further investigation in molecular detail. SAGA was first reported as a histone H3 acetyltransferase in 1997^1^, and the biological function of SAGA has been widely studied ever since then. Despite recent cryo-electron microscopy (cryo-EM) studies of Tra1 from *S. cerevisiae* (3.7 Å)^6^ and of the SAGA complex from *Pichia pastoris* (11.7 Å)^7^, the structure of an intact SAGA complex at higher resolution is needed to further elucidate its assembly and regulation.

We successfully purified an endogenous SAGA complex that contains all 19 subunits from fermented *S. cerevisiae* cells after optimization of growth conditions (Fig. 1a; Supplementary Fig. S1b). To avoid sample disassembly, the purified native SAGA complex was subject to cross-linking prior to negative stain and cryo-EM specimen preparation (Supplementary Fig. S1a, c). The structure of SAGA was determined by single-particle cryo-EM analysis at a resolution up to 6.9 Å (Fig. 1b; Supplementary Fig. S2 and Table S1). The overall shape of SAGA resembles an open padlock, and can be divided into lobes A and B (Fig. 1b, c; Supplementary Fig. S2c). To further improve the map, lobe A and lobe B were individually filtered out for focused refinement. Finally, the cryo-EM map of lobe A was reconstructed at 4.6 Å at FSC = 0.143 (Supplementary Fig. S2c, d, g). Consistent with a previous report^6^, the 3744-residue Tra1 was assigned to lobe A. After docking of the 3.7 Å free Tra1 model (PDB ID: 5OJS)^6^, the structure of Tra1 in the context of SAGA was further modeled and refined to fit into the 4.6 Å cryo-EM map. By contrast, lobe B is less well resolved, and an overall resolution of 9.3 Å was calculated at FSC = 0.5 (Supplementary Fig. S2d, f). The two enzymatic modules, HAT and DUB, are located within lobe B. The blurry density of lobe B likely reflects conformational dynamics of the catalytic modules in the absence of the nucleosomal substrate.

**Fig. 1:**
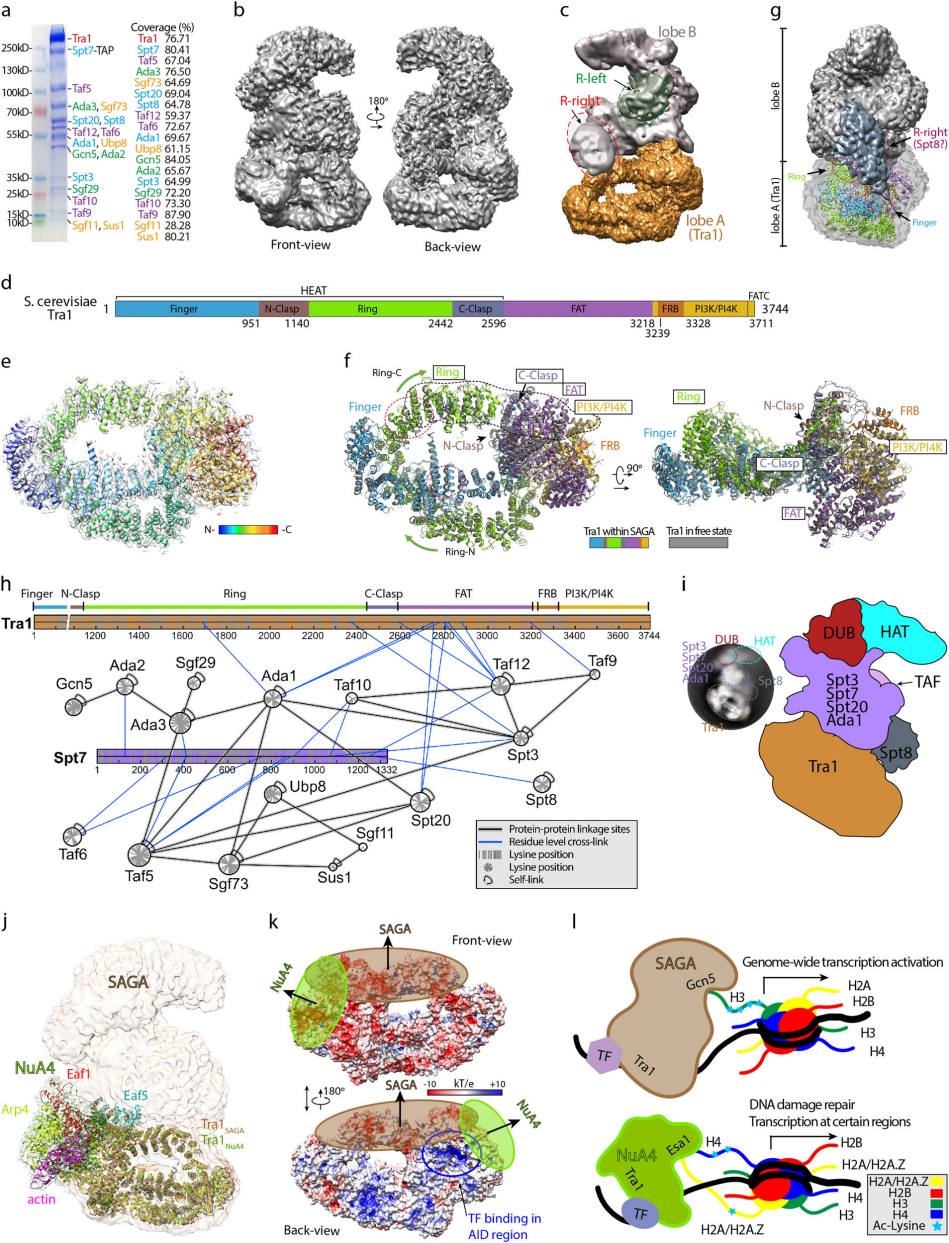
Cryo-EM structure of *S. cerevisiae* SAGA complex. **a** Coomassie blue-stained SDS–PAGE gel and mass spectrometry analysis of a purified native *S. cerevisiae* SAGA complex. Subunit names are color-coded with green for HAT, yellow for DUB, blue for SPT (red for Tra1), and purple for TAF. **b** Overall cryo-EM map of *S. cerevisiae* SAGA complex in front and back views. **c** Cryo-EM map for individually refined two lobes of *S. cerevisiae* SAGA. Dark yellow, lobe A; gray, lobe B. Two wing-like regions, R-right and R-left, were marked with dashed circles. **d** Domain architecture of *S. cerevisiae* Tra1. **e** Overall structure of Tra1 docked in its cryo-EM map. Tra1 was shown as a ribbon in rainbow color from N- to C-terminus. **f** Alignment of Tra1 in the free state (gray) and in the SAGA complex (color-coded by domains). Green arrows denote the N-terminal half of Ring (Ring-N) and the C-terminal half of Ring (Ring-C). Red dashed circles, R-right contact surfaces. **g** Low-threshold SAGA map highlighting an elongated R-right region (dark gray) that contacts Tra1. The rainbow-colored Tra1 was docked into the 50%-transparent Tra1 map. Contact surface within Ring and Finger domains is highlighted by red dashed circles. **h** CXMS analysis of the SAGA complex. The two largest subunits, Tra1 and Spt7, are shown as a spreaded domain architecture. Red and black curved lines indicate inter or intra self-links, respectively. Blue and black straight lines indicate protein–protein cross-links. **i** Cartoon model of the SAGA assembly. A 2D class average image is shown on the left. The SAGA modules or subunits are color-coded as indicated. **j** Superimposition of NuA4 and SAGA by aligning the two Tra1 structures (Tra1_SAGA_, dark yellow; Tra1_NuA4_, green). The subunits of NuA4 (PDB ID: 5Y81)^9^ are color-coded as indicated. **k** Electrostatic surface view of Tra1. Electrostatic potential is expressed as a spectrum ranging from –10 kT/e (red) to + 10 kT/e (blue). Buried surfaces upon SAGA or NuA4 complex formation are shaded by ovals. Putative transcription factor (TF)-binding site is marked with a blue circle. AID activator interaction domain^4^. **l** Working model for TF-facilitated chromatin targeting of SAGA and NuA4

Tra1 is composed of HEAT (Finger, N-clasp, Ring, and C-clasp), FAT, FRB, PI3K/PI4K, and FATC domains from the N- to the C-terminus (Fig. 1d). We eventually generated an alanine-substituted structure of Tra1 consisting of alanine, glycine, and proline residues (Fig. 1e). Tra1 is characteristic of a helical solenoid fold and arranged into a “θ”-like structure, in which a Ring motif (1140–2442) within the HEAT domain forms the ring (Fig. 1f). Tra1 functions as a base to organize other subunits in lobe B of SAGA (Fig. 1c). Structural comparison of free Tra1 with its complex state revealed only regional conformational adjustments upon SAGA assembly, which reflects the structural rigidity of the solenoid fold of Tra1 (Fig. 1f; Supplementary Movie S1).

A comparison of the cryo-EM map of *S. cerevisiae* SAGA with the reported one from *P. pastoris*^7^ revealed two better-defined regions in lobe B, referred to as R-right and R-left regions (Fig. 1b, c; Supplementary Fig. S3). The R-right region is located at the middle of the SAGA next to Tra1. Under lower-threshold levels, the density of the R-right region extends from the C-terminal region of Ring (Ring-C) (~2130–2266) toward the middle region of Finger (~579–691) of the HEAT domain, thus spanning over Tra1 like an arch (Fig. 1f, g; Supplementary Fig. S3a, b). In addition, the elongated ridge surface formed by FAT, PI3K/PI4K, C-Clasp, and Ring-C of Tra1 constitutes the major contact interface with lobe B (Fig. 1f). Previous negative staining studies of Spt8-GFP SAGA suggested positioning of Spt8 next to Tra1 (Supplementary Fig. S4a)^8^. Direct comparison of our 2D class average images (both negative staining and cryo-EM) with the reported one of Spt8-GFP suggests that Spt8 is likely located to the R-right region (Fig. 1g; Supplementary Fig. S4b).

To further dissect the subunit organization of lobe B, we next performed chemical cross-linking of proteins coupled with mass spectrometry (CXMS) analyses. In total, 139 unique inter- and 184 unique intra-subunit cross-linking pairs were identified (Fig. 1h; Supplementary Table S2), which reflects an extensive interconnectivity among SAGA subunits. Direct cross-linking of Tra1 with Taf12, Ada1, Spt20, Spt3, and Taf9 indicated that these subunits in lobe B are located next to Tra1 (Fig. 1h). Remarkably, high-confidence cross-linking peaks suggest that Taf12/Ada1/Spt20/Spt3 are clustered to contact with Tra1 through a surface formed by Ring-C, C-Clasp, and FAT (Fig. 1h; Supplementary Figs. 5–7). Aided by GFP tracing, the HAT (Gcn5, Ada2, Ada3, and Sgf29) and DUB (Ubp8, Sgf11, Sgf73, and Sus1) modules have been previously shown to locate at the Tra1-distal end of lobe B^8^. Consistently, few cross-linking pairs were detected between Tra1 and DUB/HAT (Fig. 1h).

The second largest subunit, Spt7, plays a central role in lobe B organization, as reflected by its broad interactions with most components from SPT (Spt3 and Spt8), TAF (Taf5, Taf6, and Taf12), and HAT (Ada2 and Ada3) modules (Fig. 1h). Meanwhile, few cross-linking pairs were detected between Tra1 and Spt7/Taf5/Taf6/Taf10, suggesting that these subunits are clustered in the middle part of lobe B with Spt7 positioned at the core. Unlike Taf5, Taf9, Taf10, and Taf12 that are cross-linked to multiple subunits, Taf6 is rather isolated and mainly cross-linked to Spt7 (similar to Spt8, ~66.2 kDa; Fig. 1h). Combining with our EM analysis, Taf6 (~58 kDa) is probably located to the relatively exposed R-left region. This is consistent with previous reports in which the “R-left” region was assigned as part of TAF^8^. Collectively, we proposed an assembly model of SAGA (Fig. 1i; Supplementary Figs. S4b, S8a), which lays out a structural framework for functional analysis of the SAGA holo enzyme.

SAGA and NuA4 are two major acetyltransferases in yeast and share the largest subunit Tra1. To compare the role of Tra1 in organizing SAGA and NuA4, we superimposed the two complexes by aligning Tra1 together (Fig. 1j). Distinct surfaces of Tra1 were involved in the assembly of SAGA and NuA4, and the rest parts of the two enzymatic machineries are built up in perpendicular directions (Fig. 1k). Notably, NuA4 extends from the lateral surface of the Tra1 ring, while the SAGA grows up from the central surface of Tra1. We noticed that the Eaf5 subunit of NuA4 clashes with lobe B of SAGA (Fig. 1j)^9^, which suggests that SAGA and NuA4 unlikely function as one super-complex, unless an event of subunit switch occurs.

SAGA serves as a general cofactor in yeast, genome-widely linking epigenetic modifications to transcriptional activation in concert with sequence-specific TFs. We next performed surface charge analysis of the reported 3.7 Å Tra1 cryo-EM structure (PDB ID: 5OJS)^6^ in the context of SAGA (Fig. 1k; Supplementary Fig. S8). Intriguingly, the exposed Tra1 surface upon complex formation displayed bipolarized electrostatic potential distribution patterns, in which the front-view surface is mainly negatively charged and the back-view surface is largely positively charged (Fig. 1k). The positively charged back-view surface of Tra1 is likely involved in binding with DNA or the acidic activation domain of TFs, hence orchestrating physical TF recruitment, cooperative DNA binding, and histone modifications, in particular nuclear processes regulated by SAGA or NuA4 (Fig. 1l). In support, the Vp16/Gal4 activator interaction domain (AID) of Tra1 has been mapped to segment 2233–2836 that spans Ring-C to FAT in a previous report (Fig. 1e, k)^4^.

Histone H3 acetylation and ub-H2B deubiquitylation are two tightly coupled activities of SAGA, which raises the question of how the HAT and DUB modules are coordinated during transcription. Hence, we docked ub-nucleosome-DUB structure into the SAGA (PDB ID: 4ZUX, Supplementary Fig. S9)^10^, which might constitute one of the possible intermediate states for nucleosome recognition by SAGA. Intriguingly, a ubiquitylated nucleosome was captured by the DUB module and swung away from the HAT module. It appears that the HAT and DUB modules could not engage in the catalysis of one nucleosome substrate concurrently. Our structural analysis suggests that the DUB and HAT modules of SAGA likely act sequentially, in which histone H3 acetylation by HAT is preceded by the ubiquitin removal and ub-nucleosome recognition by DUB. Such a sequential catalysis model awaits further complex structural studies.

## Accession codes

The electron density maps for *S. cerevisiae* full SAGA complex and subunit Tra1 have been deposited in EMDB under accession codes EMD-9663 and EMD-9664, respectively. The atomic coordinates for a pseudo-structure of Tra1 have been deposited in the Protein Data Bank under accession code 6IG9.

## Supplementary information

Supplementary_Materials

Supplementary Table S2

Supplementary Movie S1

## Supplementary information

**Supplementary Information** accompanies the paper at (10.1038/s41421-019-0094-x).
