# Supplementary material for: Architecture of Saccharomyces cerevisiae SAGA complex
Source: Cell Discov. 2019 May 7;5:25. doi: 10.1038/s41421-019-0094-x (PMC6502868; doi:10.1038/s41421-019-0094-x)
Supplement: Supplementary file 1 — Supplementary_Materials [file 41421_2019_94_MOESM1_ESM.pdf]

Supplementary information

Architecture of *Saccharomyces cerevisiae* SAGA Complex

Gaochao Liu[#1], Xiangdong Zheng[#1], Haipeng Guan[#1], Yong Cao[2], Hongyuan Qu[1], Junqing Kang[1], Xiangle Ren[1], Jianlin Lei[1], Meng-Qiu Dong[2], Xueming Li[1], and Haitao Li[*1]

[1]MOE Key Laboratory of Protein Sciences, Beijing Advanced Innovation Center for Structural Biology, Tsinghua-Peking Joint Center for Life Sciences, School of Life Sciences and School of Medicine, Tsinghua University, Beijing 100084, China

[2]National Institute of Biological Sciences, Beijing 102206, China

[#]These authors contributed equally to this work

[*]Correspondence should be addressed to HL (lht@tsinghua.edu.cn)

Supplementary information, Materials and Methods

Yeast strain construction

Saccharomyces cerevisiae BY4741 (*MATa his3Δ1 leu2Δ0 met15Δ0 ura3Δ0*) was used to construct all the strains in this study following the method previously reported[1,2]. In brief, for Spt7-TAP strain construction, the C-terminal TAP tag followed by the URA3 selectable marker was PCR-amplified from pBS1539 plasmid using yeast homologous sequence containing primers, and then introduced to yeast genome for *in vivo* homologous recombination. Yeast transformation was performed using a lithium acetate procedure[3], and positive colonies were confirmed by PCR reactions followed by sequencing.

Yeast fermentation

To harvest a large quantity of cell pellets, Spt7-TAP yeast cells were grown in a 100 l New Brunswick BioFlo 610 fermenter (Eppendorf) at 30 °C. In detail, three Spt7-TAP yeast colonies were picked and grew overnight by shaking in 50 mL YPD medium. The overnight culture was then used to inoculate 80 L YPD medium. Until OD_{600} of the culture reached seven, 4 liters of 20% w/v glucose solution was then supplemented, allowing cells to grow rapidly. After two hours, yeast cells were collected using a CR22N high-speed refrigerated centrifuge (Hitachi Koki). The harvested cells were washed once with PBS, and then resuspended in one-quarter pellet volume of lysis buffer (40 mM HepesNa, pH 7.4, 350 mM NaCl, 10% glycerol, 0.1% IGEPAL CA-630, 1 mM PMSF, complete EDTA-free protease inhibitor cocktail (Roche), PhosSTOP phosphatase inhibitor cocktail (Roche)). Cell suspension was dropped into liquid nitrogen to form small frozen beads prior to storing at –80°C.

For small-scale fermentation in flasks, the overnight culture was inoculated into 1 L YPD medium, and grown at 30°C until OD_{600} reached four. 50 mL of 20% (w/v) glucose solution was then supplemented to allow yeast cells to grow rapidly. After two hours, yeast

cells were collected by centrifuge at 4000 rpm for 15 min. Cell pellets were resuspended and frozen as described above.

SAGA complex purification

Frozen yeast cell beads were ground into powder using a Type GT200 grinder (Beijing Grinder Instruments, China). The frozen cell powder was resuspended in one-quarter pellet volume of lysis buffer, and then clarified by centrifuge at 33,000 × *g* for 30 min. Native SAGA complex was purified from the clarified supernatant following the procedures described[4] with minor modifications. In particular, the supernatant was incubated with IgG Sepharose-6 Fast Flow resin (GE Healthcare) at 4 °C for 3 h. The resin was then washed with lysis buffer and resuspended in a total of 3 ml of TEV-C buffer (40 mM HepesNa, pH 7.4, 150 mM NaCl, 10% glycerol, 0.1% IGEPAL CA-630). SAGA complex was eluted by tobacco etch virus protease cleavage at 18°C for 1.5 h. 2 μL of benzonase nuclease (Sigma-Aldrich) was added to the eluent and incubated on ice for 30 min. 500 μL of the nuclease-treated SAGA sample was overlaid on one linear 15-45% glycerol gradient. The other sample was evenly overlaid on five 15-45% glycerol gradients containing 0.0-0.1% glutaraldehyde. GraFix[5] was performed by ultracentrifuge at 32,800 rpm for 16 h at 4 °C, and all of the six gradients were fractionated every 500 μL. Uncross-linked fractions were TCA-precipitated and analyzed by silver staining SDS-PAGE. Corresponding fractions that contained cross-linked SAGA were pooled together and dialyzed against dialysis buffer (40 mM HepesNa, pH 7.4, 150 mM NaCl, 2 mM Tris-Cl, 2 mM DTT) overnight. After dialysis, the sample was concentrated and loaded onto a Superose 6 5/150 column (GE Healthcare). SAGA containing fractions were then combined and concentrated for grid preparation.

Specimen preparation and data acquisition

For negative staining, 4 μl of the sample at a concentration of ~0.02 mg/mL were applied onto a glow-discharged continuous lacey carbon grid (Beijing Zhongjingkeyi Technology,

China) for 1 min, and then the grid was washed in uranyl acetate (2% w/v) for 30 s prior to drying. Images were taken on a FEI Tecnai Spirit Bio TWIN microscope operating at 120 kV.

For cryo-EM, an aliquot of 4 μl of the sample at a concentration of ~0.2 mg/mL were applied to a glow-discharged grid (Quantifoil 1.2/1.3), which is coated beforehand with a ~5-nm homemade continuous carbon support layer. Then the grid was blotted for 3.5 s, and plunged into liquid ethane cooled by liquid nitrogen.

The EM grids was imaged on a Cs-corrector-equipped FEI Titan Krios electron microscope, operating at 300 kV with a nominal magnification of 81,000 ×. Images were then recorded by a Gatan K2 Summit detector using the super-resolution mode, with a pixel size of 1.401/2=0.7005 Å. AutoEMation[6] was used for fully automated data collection. Defocus was also set automatically, and the values varied from -1.2 to 3.0 μm. The total exposure time was 8.0 s, and each micrograph was dose-fractionated to 32 frames with a dose rate of ~5.6 e^{-}/s/$Å^{2}$. All 32 frames in each stack were aligned firstly using dosefgpu_driftcorr and Fourier-binned to a pixel size of 1.401 Å due to limited storage. These binned stacks were corrected in both whole-image and local-region using MotionCor2[7]. The defocus value of each image was determined by Gctf[8]. Finally, A total of 8,526 cryo-EM micrographs were collected.

Image processing and 3D reconstruction

Briefly, a randomly selected image subset (200 images) was auto-picked using Gautomatch (http://www.mrc-lmb.cam.ac.uk/kzhang/Gautomatch/), and then a single run of unsupervised 2D classification by Relion-2[9,10] was followed to generate a 2D reference for large scale particle auto-picking. In total, ~1.35M particles were generated from ~8200 good images using reference-based auto-picking mode in Gautomatch. This dataset was further cleaned by 2D classification in cryoSPARC[11] and 1.2M particles were remained. Then, a single run of 3D classification was performed on the 2× binned particles (pixel size: 2.802 Å) with the 60 Å low-passed SAGA map (generated by cryoSPARC[11]) as the initial reference, which yielded one best class (24.65%) after 75 iterations using Relion-2. Next, ~295,800 particles

were re-extracted, re-centered, re-grouped, and used for refinement to converge into a map with a reported resolution of 8.5 Å. Random-phase 3D classification (based on Relion-1.4, developed by Dr. Qian Zhou) [12] was performed to further clean the heterogeneous dataset, which yielded a subset of 176,464 particles. Next, 3D refinement and reconstruction were conducted to converge into a map with a reported resolution of 8.4 Å, then post-processed at 6.9 Å. Focused refinements of Tra1 subunit with local masks were performed subsequently, leading to an improved Tra1 region reported at 4.6 Å. For the lobe-B, the Tra1 subtracted dataset was generated by the subtraction module in Relion-2, and then directly refined and reconstructed at the local search mode which yielded an 8.2 Å map.

Reported resolutions were calculated on the basis of the golden-standard FSC 0.143 criterion, and the FSC curves were corrected for the effects of a soft mask on the FSC curve using high-resolution noise substitution[13]. All the visualization and evaluation of the map was performed within UCSF Chimera[14], and the local resolution map was calculated using ResMap[15]. Statistical information is shown in **Supplemental information, Table S1**.

Model building and refinement

The available 3.7 Å resolution cryo-EM structure of *S. cerevisiae* Tra1 (PDB ID: 5OJS)[16] was processed using the Chainsaw program[17] in CCP4 to generate the poly-alanine pseudo-model. The initial cryo-EM map was auto-sharpened using phenix.auto_sharpen[18] implemented in PHENIX[19]. The poly-alanine pseudo-model was subsequently aligned into an auto-sharpened density map in Coot[20]. Model building in Coot was conducted in several aspects, for which unfitted regions were appropriately adjusted using rigid-body fitting and real-space refinement, and missed loops were *de novo* built manually. Prolines and glycines were reserved in the model without alanine-substitution. The model was refined against the auto-sharpened map using phenix.real_space_refine[21] implemented in PHENIX. The geometry statistics of the models were generated using MolProbity[22]. All the figures were prepared in PyMol (http://www.pymol.org/) or UCSF Chimera.

Chemical cross-linking of proteins coupled with mass spectrometry (CXMS)

The SAGA protein sample was divided into four aliquots of approximately 5 µg each and cross-linked with BS^3, DSS, formaldehyde and BS^3, or formaldehyde and DSS. Cross-linking with BS^3 or DSS alone was conducted as previously described[23]. Cross-linking with formaldehyde first and then BS^3 or DSS was inspired by Robinson et al[24]. Briefly, formaldehyde was added at one-tenth of the molar concentration of the lysine residues in the SAGA complex for 15-min fixation on ice, before the sample was further cross-linked with BS^3 or DSS as described[23]. All the samples were quenched by 20 mM NH_4HCO_3. Proteins were precipitated with ice-cold acetone, resuspended in 8 M urea, 100 mM Tris, pH 8.5, and treated with 5 mM TCEP at 55 °C for 30 min to reduce and denature proteins, and to reverse formaldehyde cross-links. Then, the proteins were alkylated with 50 mM iodoacetamide at RT for 20 min, diluted to 2 M urea, 100 mM Tris, pH 8.5, and digested with 0.1 µg trypsin (Promega) for 12 h at 37 °C. LC-MS/MS analyses were performed on an Easy-nLC 1000 II HPLC (Thermo Fisher Scientific) coupled to a Q-Exactive HF mass spectrometer (Thermo Fisher Scientific). Peptides were loaded on a pre-column (75 µm ID, 6 cm long, packed with ODS-AQ 10 µm, 120 Å beads from YMC Co., Ltd.) and further separated on an analytical column (75 µm ID, 12 cm long, packed with C18 1.8 µm, 100 Å resin from Welch Materials) using a linear reverse-phase gradient from 98% buffer A (0.1% formic acid in H_2O) to 28% buffer B (0.1% formic acid in acetonitrile) in 71 min at a flow rate of 220 nL/min. The top 15 most intense precursor ions from each full scan (resolution 60,000) were isolated for HCD MS2 (resolution 15,000; normalized collision energy 27) with a dynamic exclusion time of 30 s. Precursors with 1+, 2+, 7+ or above, or unassigned charge states, were excluded. Each sample was analyzed twice, and the two technical repeats were combined for data analysis. pLink software[25] was utilized to identify cross-linked peptides with precursor mass accuracy at 20 ppm, fragment ion mass accuracy at 20 ppm, and the results were filtered by applying a 5% FDR cutoff at the spectral level and then an E-value cutoff at 0.001. A

minimum of three MS/MS spectra were required for each cross-link identification. A cross-link network diagram was prepared using xiNET[26].

References

1 Puig, O. *et al.* The tandem affinity purification (TAP) method: a general procedure of protein complex purification. *Methods* **24**, 218-229 (2001).

2 Longtine, M. S. *et al.* Additional modules for versatile and economical PCR-based gene deletion and modification in Saccharomyces cerevisiae. *Yeast* **14**, 953-961 (1998).

3 Gietz, D., St Jean, A., Woods, R. A. & Schiestl, R. H. Improved method for high efficiency transformation of intact yeast cells. *Nucleic Acids Res* **20**, 1425 (1992).

4 Setiaputra, D. *et al.* Conformational flexibility and subunit arrangement of the modular yeast Spt-Ada-Gcn5 acetyltransferase complex. *The Journal of biological chemistry* **290**, 10057-10070 (2015).

5 Stark, H. GraFix: stabilization of fragile macromolecular complexes for single particle cryo-EM. *Methods in enzymology* **481**, 109-126 (2010).

6 Lei, J. & Frank, J. Automated acquisition of cryo-electron micrographs for single particle reconstruction on an FEI Tecnai electron microscope. *J Struct Biol* **150**, 69-80 (2005).

7 Zheng, S. Q. *et al.* MotionCor2: anisotropic correction of beam-induced motion for improved cryo-electron microscopy. *Nature methods* **14**, 331-332 (2017).

8 Zhang, K. Gctf: Real-time CTF determination and correction. *J Struct Biol* **193**, 1-12 (2016).

9 Kimanius, D., Forsberg, B. O., Scheres, S. H. & Lindahl, E. Accelerated cryo-EM structure determination with parallelisation using GPUs in RELION-2. *eLife* **5**, e18722 (2016).

10 Scheres, S. H. A Bayesian view on cryo-EM structure determination. *Journal of molecular biology* **415**, 406-418 (2012).

11 Punjani, A., Rubinstein, J. L., Fleet, D. J. & Brubaker, M. A. cryoSPARC: algorithms for rapid unsupervised cryo-EM structure determination. *Nature methods* **14**, 290-296 (2017).

12 Gong, X. *et al.* Structural Insights into the Niemann-Pick C1 (NPC1)-Mediated Cholesterol Transfer and Ebola Infection. *Cell* **165**, 1467-1478 (2016).

13 Chen, S. *et al.* High-resolution noise substitution to measure overfitting and validate resolution in 3D structure determination by single particle electron cryomicroscopy. *Ultramicroscopy* **135**, 24-35 (2013).

14 Pettersen, E. F. *et al.* UCSF Chimera--a visualization system for exploratory research and analysis. *J Comput Chem* **25**, 1605-1612 (2004).

15 Kucukelbir, A., Sigworth, F. J. & Tagare, H. D. Quantifying the local resolution of cryo-EM density maps. *Nature methods* **11**, 63-65 (2014).

16 Diaz-Santin, L. M., Lukoyanova, N., Aciyan, E. & Cheung, A. C. Cryo-EM structure of the SAGA and NuA4 coactivator subunit Tra1 at 3.7 angstrom resolution. *eLife* **6**, e28384 (2017).

17 Stein, N. CHAINSAW: a program for mutating pdb files used as templates in molecular replacement. *Journal of Applied Crystallography* **41**, 641–643 (2008).

18 Afonine, P. V. *et al.* New tools for the analysis and validation of Cryo-EM maps and atomic models. *bioRxiv*, doi:10.1101/279844 (2018).

19 Adams, P. D. *et al.* PHENIX: a comprehensive Python-based system for macromolecular structure solution. *Acta crystallographica. Section D, Biological crystallography* **66**, 213-221 (2010).

20 Emsley, P. & Cowtan, K. Coot: model-building tools for molecular graphics. *Acta crystallographica. Section D, Biological crystallography* **60**, 2126-2132 (2004).

21 Afonine, P. V., Headd, J. J., Terwilliger, T. C. & Adams, P. D. New tool: phenix.real_space_refine. *Computational Crystallography Newsletter* **4**, 43-44 (2013).

22 Chen, V. B. *et al.* MolProbity: all-atom structure validation for macromolecular crystallography. *Acta crystallographica. Section D, Biological crystallography* **66**, 12-21 (2010).

23 Ding, Y. H. *et al.* Increasing the Depth of Mass-Spectrometry-Based Structural Analysis of Protein Complexes through the Use of Multiple Cross-Linkers. *Analytical chemistry* **88**, 4461-4469 (2016).

24 Robinson, P. J. *et al.* Structure of a Complete Mediator-RNA Polymerase II Pre-Initiation Complex. *Cell* **166**, 1411-1422 e1416 (2016).

25 Yang, B. *et al.* Identification of cross-linked peptides from complex samples. *Nature methods* **9**, 904-906 (2012).

26 Combe, C. W., Fischer, L. & Rappsilber, J. xiNET: cross-link network maps with residue resolution. *Mol Cell Proteomics* **14**, 1137-1147 (2015).

Supplementary Figure S1

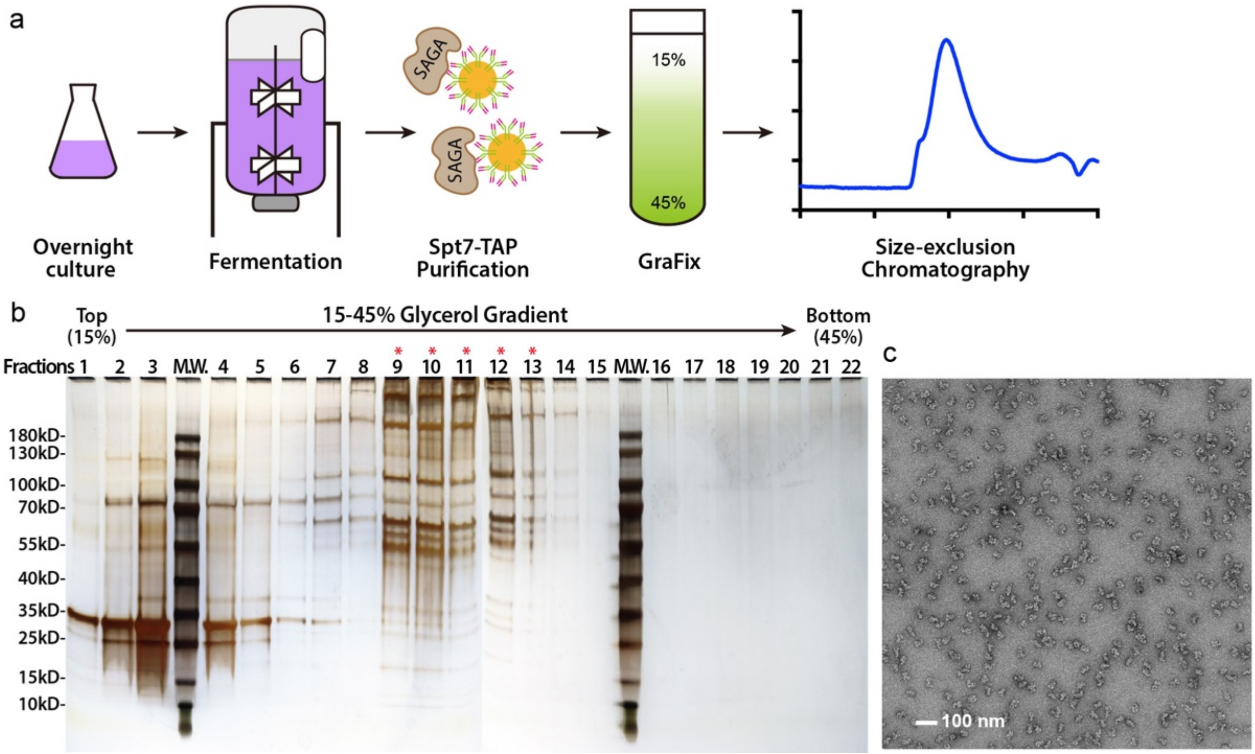

Fig. S1 Purification and negative stain of *S. cerevisiae* SAGA.

a Schematic procedure for purification of endogenous SAGA complex from *S. cerevisiae*. **b** Silver staining SDS-PAGE of uncross-linked SAGA sample purified by GraFix[5]. Red stars indicated fractions that contain intact SAGA complex. **c** Negative staining of purified SAGA sample cross-linked by glutaraldehyde. Scale bar, 100 nm.

Supplementary Figure S2

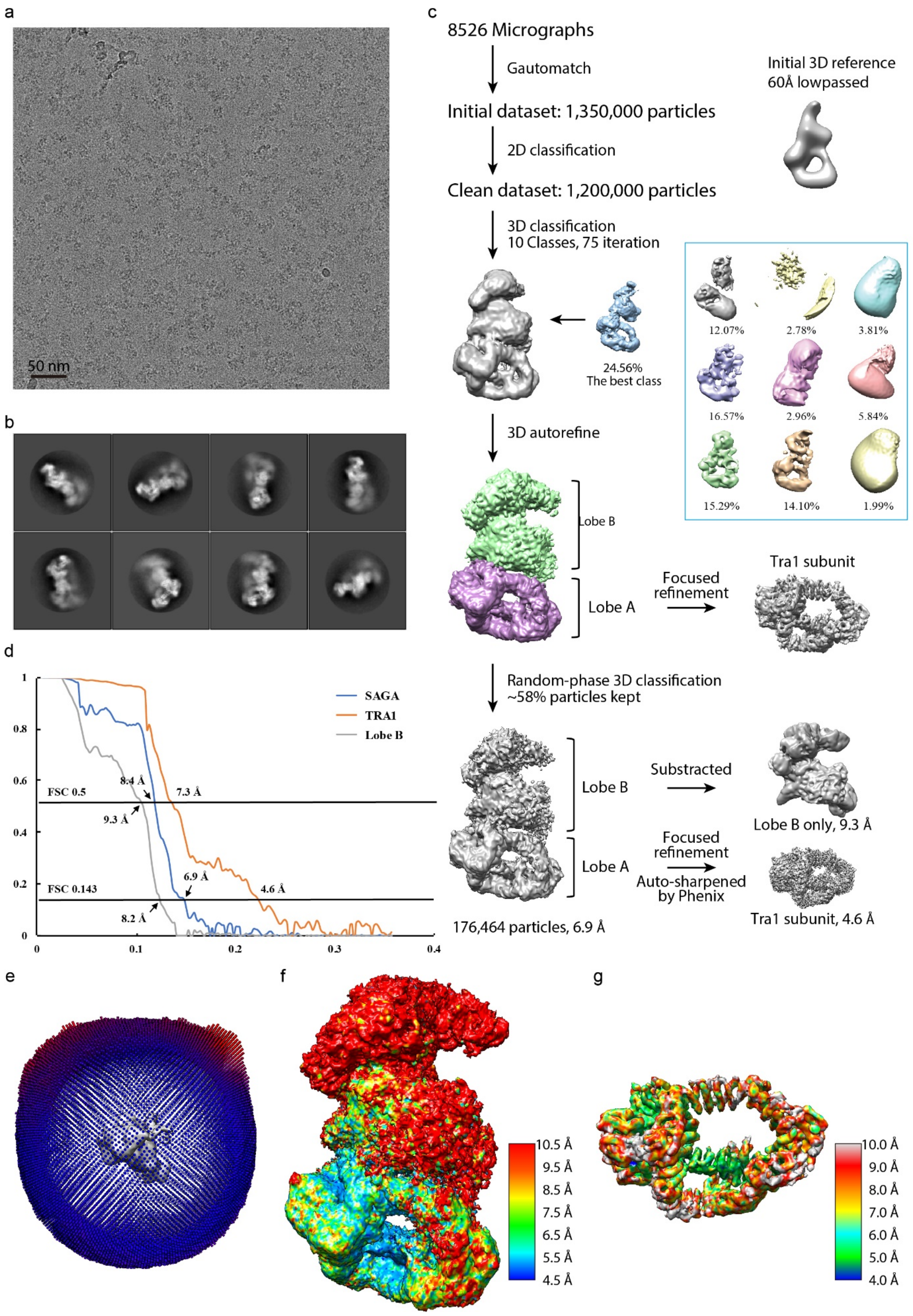

Fig. S2 Cryo-EM structure determination of *S. cerevisiae* SAGA.

a Representative cryo-EM micrograph of SAGA. **b** 2D class averages for SAGA. **c** Workflow for data collection, image processing, and reconstruction. **d** Gold-standard FSC curves of final 3D reconstructions. The resolution for lobe B was determined using FSC = 0.5 criterion. **e** Euler angle distribution of particles used in the final 3D reconstruction of SAGA. Heights of the cylinders represented the number of particles used for 3D reconstruction. **f** Local resolution map of SAGA. **g** Local resolution map of Tra1.

Supplementary Figure S3

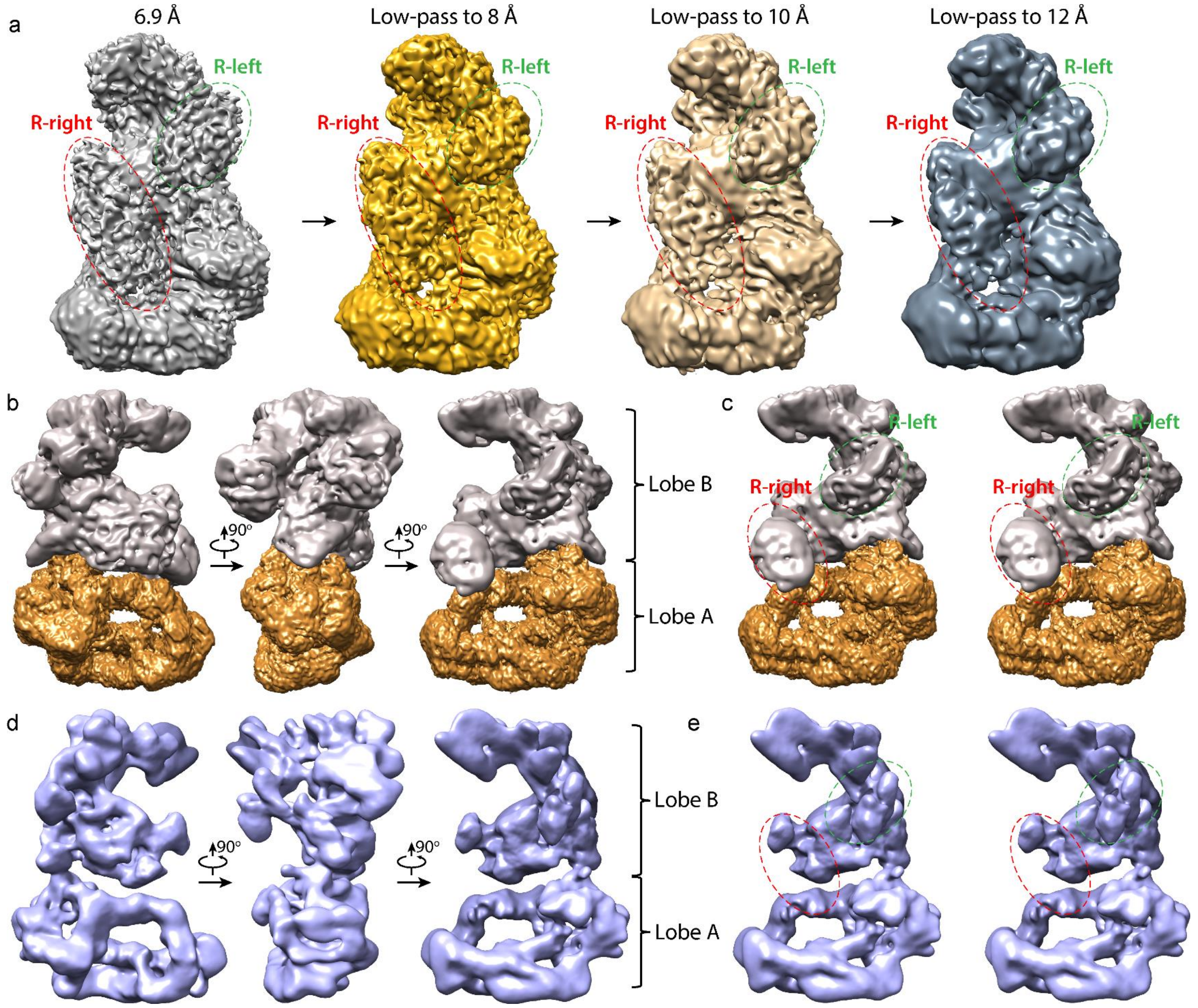

Fig. S3 Comparison of *S. cerevisiae* and *P. pastoris* SAGA cryo-EM maps.

a Low-pass filtering of *S. cerevisiae* SAGA map. R-right and R-left regions were indicated by dashed ellipses. **b** Cryo-EM map of *S. cerevisiae* SAGA. Lobe A was colored in yellowish-brown. Lobe B was colored in rosy gray. **c** Stereo pair of *S. cerevisiae* SAGA map. **d** Overall cryo-EM map of *P. pastoris* SAGA (EMDB code: EMD-3804). **e** Stereo pair of *P. pastoris* SAGA map.

Supplementary Figure S4

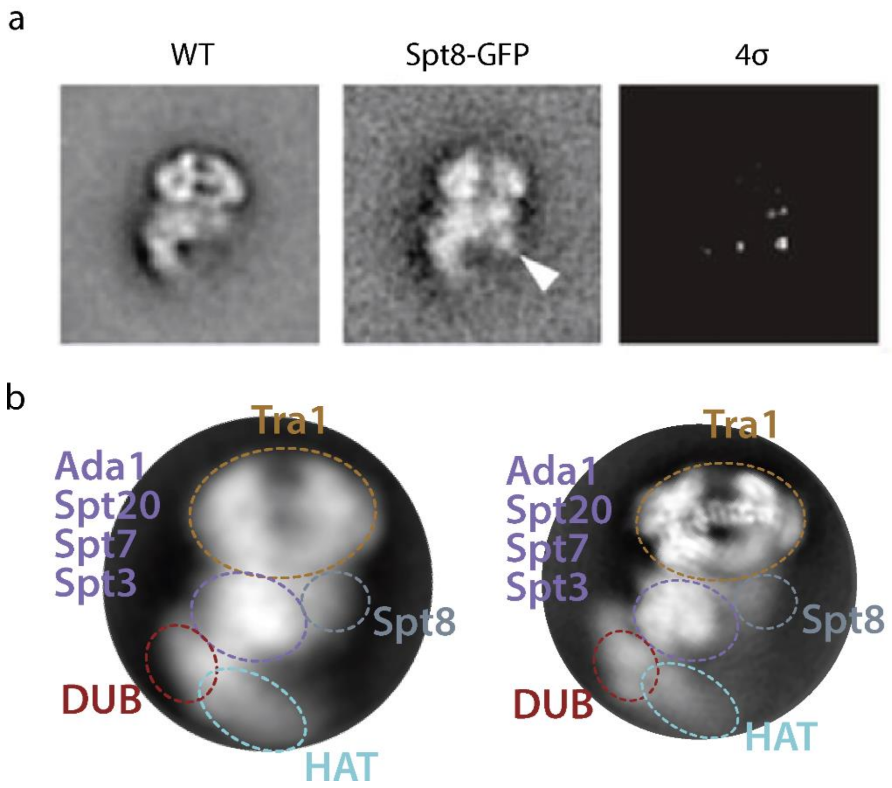

Fig. S4 Modeling the Spt8 by comparing SAGA in 2D averages with previously published GFP-tagged SAGA.

a Comparison of 2D class averages of untagged WT SAGA and GFP-tagged SAGA[4]. This is a cropped figure published on *Journal of biological chemistry* by Setiaputra D. at, al. **b** 2D class averaging images. The left panel was generated from a negative staining dataset; The right panel was generated by our cryo-EM data. The cryo-EM 2D averaging image was reflected from Fig. 1i for direct comparison.

Supplementary Figure S5

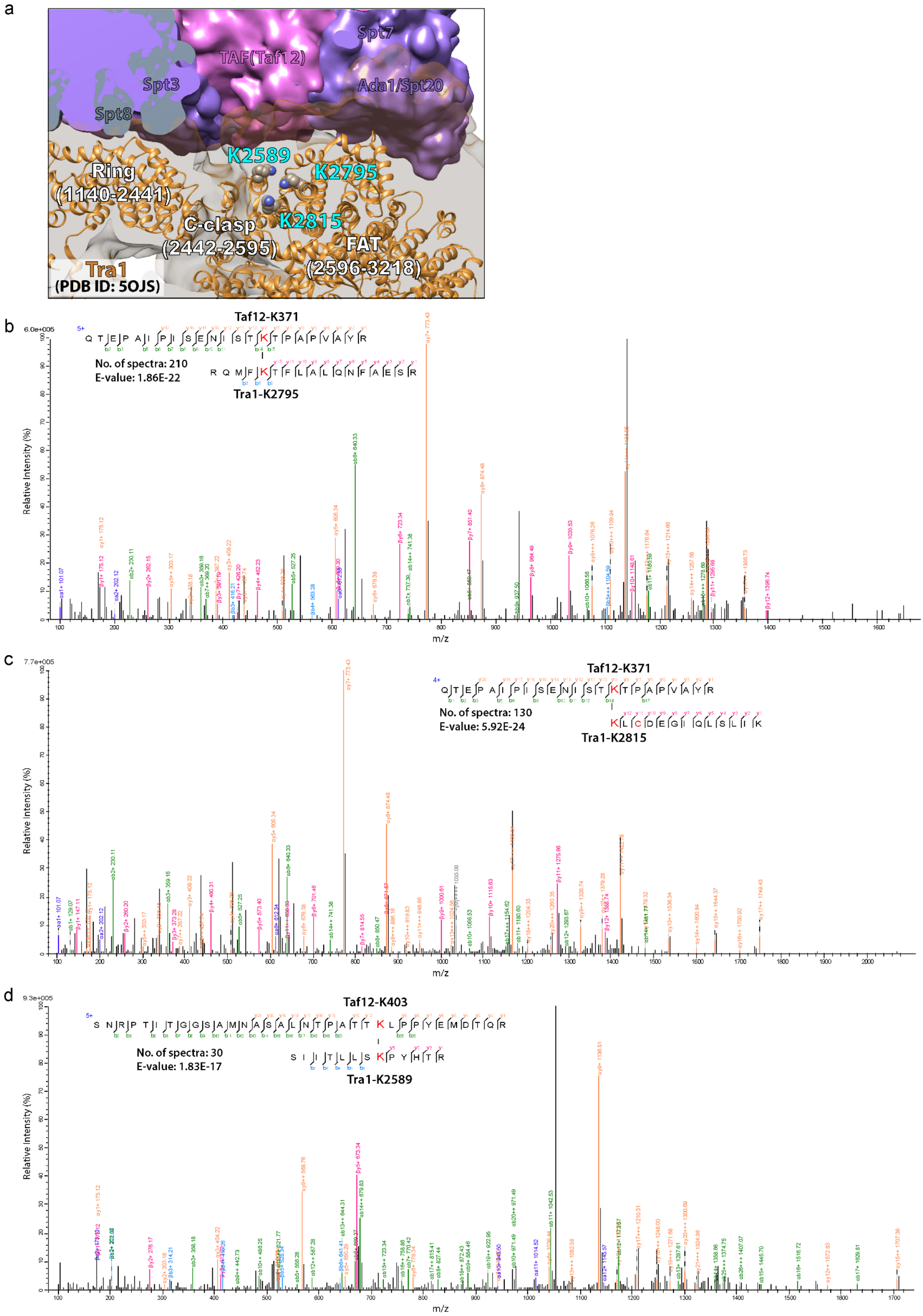

Fig. S5 Representative secondary ion mass spectra for cross-linked peptides of Tra1 and Taf12.

a Representative lysine residues on Tra1 that were cross-linked to Taf12. **b** Secondary ion mass spectrum for cross-linked peptides of $K2795_{Tra1}$-$K371_{Taf12}$. **c** Secondary ion mass spectrum for cross-linked peptides of $K2815_{Tra1}$-$K371_{Taf12}$. **d** Secondary ion mass spectrum for cross-linked peptides of $K2589_{Tra1}$-$K403_{Taf12}$.

Supplementary Figure S6

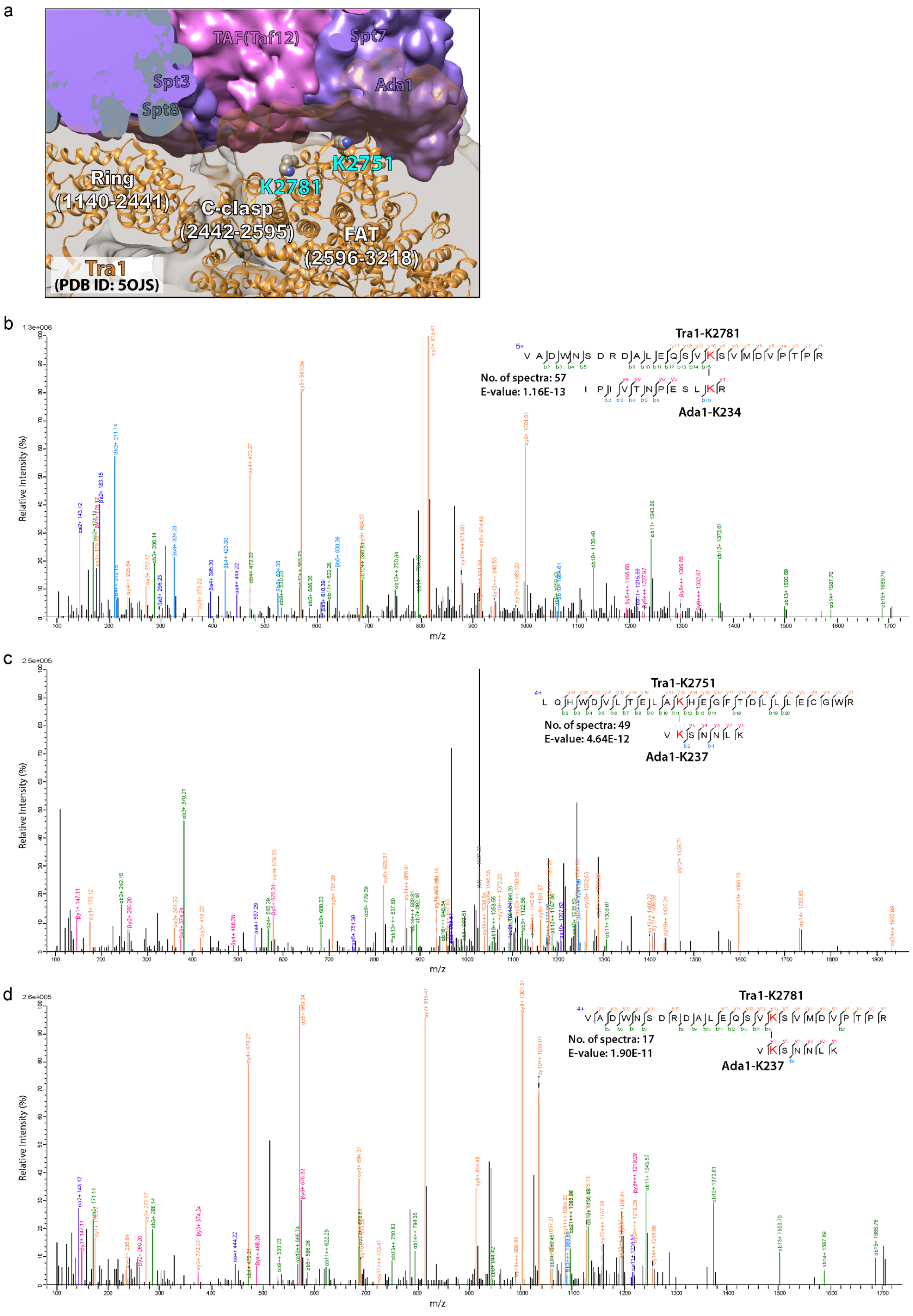

Fig. S6 Representative secondary ion mass spectra for cross-linked peptides of Tra1 and Ada1.

a Representative lysine residues on Tra1 that were cross-linked to Ada1. **b** Secondary ion mass spectrum for cross-linked peptides of $K2781_{Tra1}$-$K234_{Ada1}$. **c** Secondary ion mass spectrum for cross-linked peptides of $K2751_{Tra1}$-$K237_{Ada1}$. **d** Secondary ion mass spectrum for cross-linked peptides of $K2781_{Tra1}$-$K237_{Ada1}$.

Supplementary Figure S7

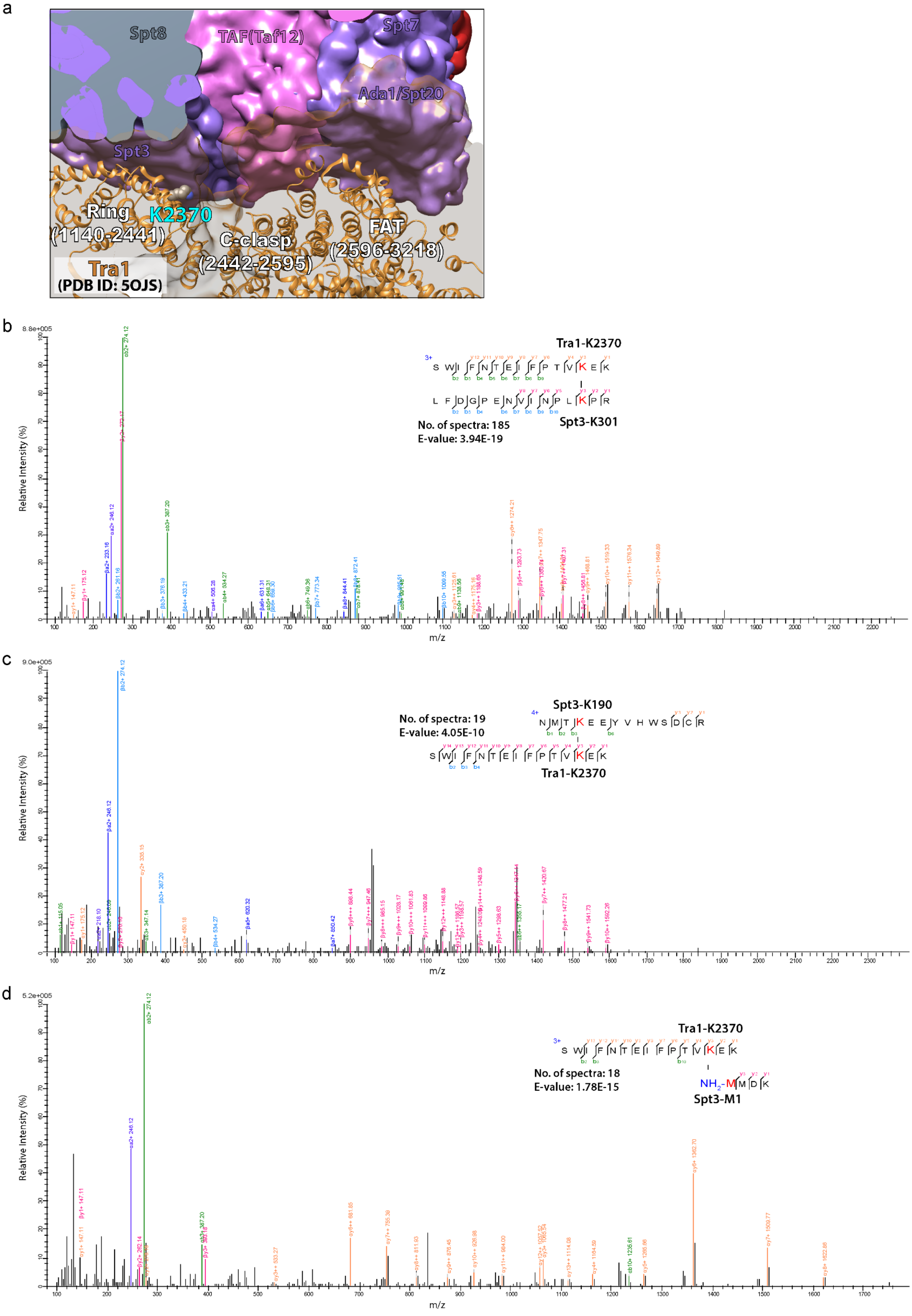

Fig. S7 Representative secondary ion mass spectra for cross-linked peptides of Tra1 and Spt3.

a Representative lysine residue on Tra1 that was cross-linked to Spt3. **b** Secondary ion mass spectrum for cross-linked peptides of $K2370_{Tra1}$-$K301_{Spt3}$. **c** Secondary ion mass spectrum for cross-linked peptides of $K2370_{Tra1}$-$K190_{Spt3}$. **d** Secondary ion mass spectrum for cross-linked peptides of $K2370_{Tra1}$-$M1_{Spt3}$.

Supplementary Figure S8

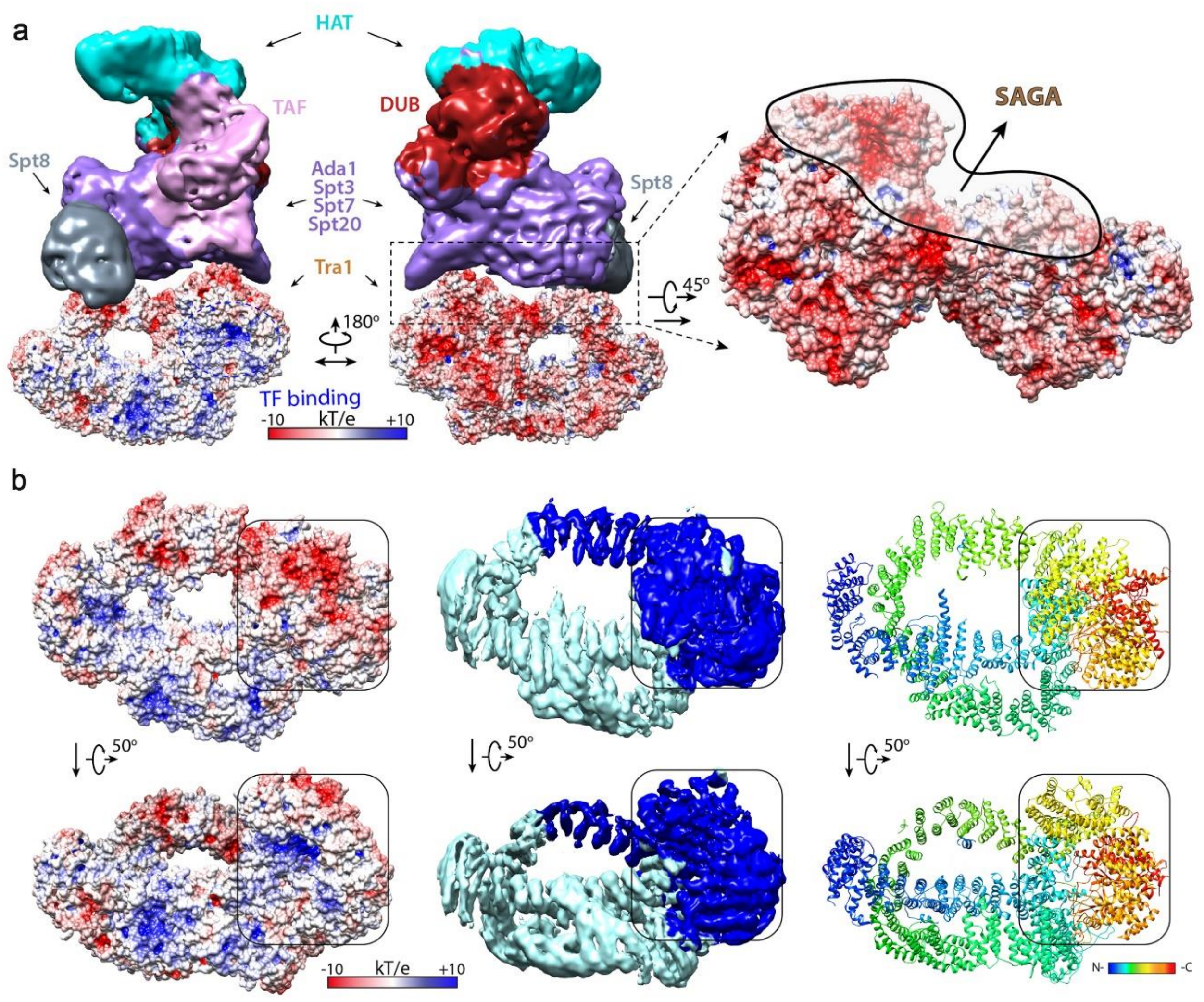

Fig. S8 An assemble model of *S. cerevisiae* SAGA, and its organization based on Tra1. **a** Docking of electrostatic potential surface of Tra1 to SAGA map. The blue dashed line indicated potential regions for transcriptional factors binding. The black rectangular dashed line outlined regions on Tra1 that were directly involved in SAGA subunit arrangement. **b** Electrostatic analysis of Tra1 potential surface. Left: Electrostatic potential surface (–10 $kT \cdot e^{-1}$, red; +10 $kT \cdot e^{-1}$, blue) of free state Tra1 (PDB ID: 5OJS). Middle: Cryo-EM map of S. cerevisiae Tra1. C-terminal Tra1 which was determined to directly interact with acidic transcription factors[27]. Right: Ribbon show of overall Tra1 structure. Black rectangles indicated the C-terminal of Tra1.

Supplementary Figure S9

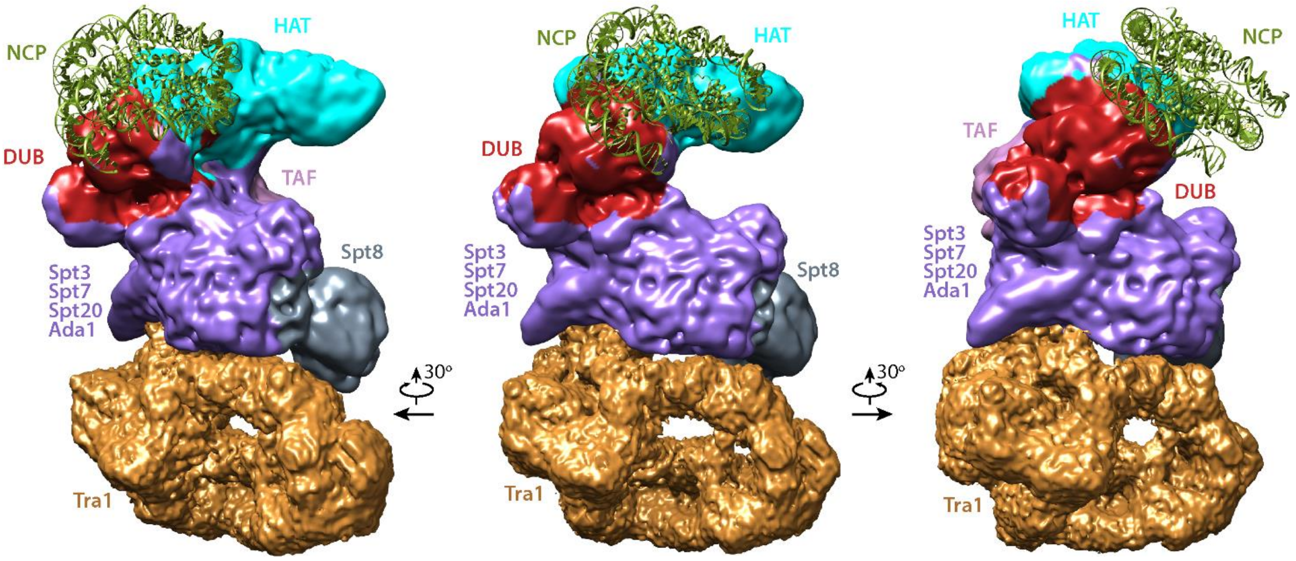

Fig. S9 Model for nucleosome recognition by SAGA.

Modelling of nucleosome structure (from PDB ID: 4ZUX) into SAGA map. Color code: HAT in cyan, DUB in red, TAF in plum, Tra1 in dark yellow, Ada1/Spt3/Spt7/Spt20 in medium purple, Spt8 in gray, nucleosome in olive.

Supplementary information, Table S1

Cryo-EM data collection and model statistics

	Overall SAGA	Tra1
Data collection		
Magnification	81,000x	81,000x
Pixel size (Å)	1.401	1.401
Defocus range (µm)	1.2-3.0	1.2-3.0
Voltage (kV)	300	300
Electron dose (e^-/Å^2)	5.6	5.6
Detector	Gatan K2	Gatan K2
Collected movie stacks	8,526	8,526
Reconstruction		
Particles for refinement	176,464	176,464
Symmetry imposed	C1	C1
Map resolution (FSC 0.5)	8.4 Å	7.3 Å
Map resolution (FSC 0.143)	6.9 Å	4.6 Å
Map sharpning bfactor (Å^2)	-275	-185
Model statistics		
Bond lengths (Å)	--	0.007
Bond angles (°)	--	1.073
MolProbity score	--	2.07
Clash score	--	9.65
Ramachandran plot statistics		
Favored (%)	--	89.66
Allowed (%)	--	10.16
Disallowed (%)	--	0.18
